# Establishing a three-dimensional scaffold model of hepatoblastoma

**DOI:** 10.3389/fbioe.2023.1229490

**Published:** 2023-11-23

**Authors:** Elena Johanna Weigl, Salih Demir, Tanja Schmid, Alina Hotes, Oliver Muensterer, Roland Kappler

**Affiliations:** Department of Paediatric Surgery, Dr. von Hauner Children’s Hospital, University Hospital, LMU Munich, Munich, Germany

**Keywords:** tumour model, biological liver scaffold, hepatoblastoma, large-scale production, three-dimensional

## Abstract

**Introduction:** Emerging technologies such as three-dimensional (3D) cell culture and the generation of biological matrices offer exciting new possibilities in disease modelling and tumour therapy. The paucity of laboratory models for hepatoblastoma (HB), the most prevalent malignant liver tumour in children, has hampered the identification of new treatment options for HB patients. We aimed to establish a reliable 3D testing platform using liver-derived scaffolds and HB cell lines that reflect the heterogeneous biology of the disease so as to allow reproducible preclinical research and drug testing.

**Methods:** In a sequence of physical, chemical and enzymatic decellularisation techniques mouse livers were stripped off all cellular components to obtain a 3D scaffold. HB cell lines were then seeded onto these scaffolds and cultivated for several weeks.

**Results:** Our newly generated biological scaffolds consist of liver-specific extracellular matrix components including collagen IV and fibronectin. A cultivation of HB cell lines on these scaffolds led to the formation of 3D tumour structures by infiltration into the matrix. Analyses of drug response to standard-of-care medication for HB showed reliable reproducibility of our stocked models.

**Discussion:** Our HB models are easy-to-handle, producible at large scale, and can be cryopreserved for ready-to-use on-demand application. Our newly generated 3D HB platform may therefore represent a faithful preclinical model for testing treatment response in precision cancer medicine.

## 1 Introduction

Hepatoblastoma is a malignant embryonal tumour predominantly diagnosed in very young children (Tulla et al., 2015). With only around 30 new cases per year in Germany, it is a rare entity, but represents two-thirds of all paediatric liver tumours (Kaatsch and Grabow, 2012; Czauderna et al., 2014). Risk factors for hepatoblastoma are extremely premature birth, a very low birth weight and genetic disposition (Tanimura et al., 1998; Tomlinson and Kappler, 2012; Ikeda and Nakamura, 2015; Yang et al., 2021). Hepatoblastoma displays varying histologies, including fetal, embryonal, and small cell undifferentiated characteristics, which are occasionally accompanied by mesenchymal components (Finegold et al., 2007). Primary resection is only feasible in small solitary masses and standard therapy includes chemotherapy and extensive surgery (Malogolowkin et al., 2011). The refinement of clinical risk stratifications alongside the development of advanced therapy regimens and improved surgical techniques has increased the 5-year survival rate up to 80% (Zsíros et al., 2010; Zsiros et al., 2013; Aronson et al., 2014). However, in high-risk patients with metastatic or treatment-resistant hepatoblastoma, survival rates remain significantly lower (Perilongo et al., 2009; Zsíros et al., 2012; Zsiros et al., 2013). Identifying new therapeutic approaches is essential for these patients, but has been hampered by the lack of appropriate models that closely mimic the disease. Current preclinical investigations include the use of cell lines, animal models, and tumour organoids. Rapid cell growth, reliable reproducibility and practicability are the advantages of exploring new therapy options in cell lines, but as they tend to show an increased cell growth, the success of therapies is easily overestimated (Andreotti et al., 1994; Fernando et al., 2006; Yamada and Cukierman, 2007; Cree et al., 2010). Moreover, long-term propagation of cells in two-dimensional cultures can lead to a loss of characteristic mutations and molecular features (Gazdar et al., 2010). Animal models are used to mimic the microenvironment of tumour cells in an organism with cell-extracellular matrix (ECM) interactions (Céspedes et al., 2006; Man et al., 2007; Rikhi et al., 2016). However, in addition to the ethical issues of animal models, these are time and cost consuming and results are rarely transferable to humans (Hackam and Redelmeier, 2006; Perel et al., 2007). Spheroids and tumour organoids have emerged as three-dimensional (3D) alternatives, allowing cell-cell and cell-ECM interactions, biochemical gradients, organ-like architecture and functionality (Huch et al., 2017). Organoids are organ-like structures derived from adult precursor cells, embryonal or pluripotent stem cells and cultivated in gels containing ECM components such as Matrigel or Basement Membrane Extract (Huch et al., 2015). Unfortunately, the cultivation of tumour organoids faces the challenge of reproducibility and takes several months (Huch et al., 2013; Huch et al., 2017; Saltsman et al., 2020). The aim of this work was to establish an easy to use, cost- and time-effective, 3D hepatoblastoma model to bridge the gap between tumour cell lines, animal models and organoids and to generate a reliable preclinical testing platform that can be stored until needed.

## 2 Materials and methods

### 2.1 Generation of a decellularised liver matrix (DLM)

Livers were resected from 6 month old male and female C57BL/6 mice. Animals were kept following the National regulations and guidelines for the Care and Use of Laboratory Animals. The studies involving animals were reviewed and approved by the Regierung von Oberbayern, Munich with approval number ROB-55.2-2532.Vet_02-19-63. Both the inferior vena cava and the portal vein were immediately cannulated with a 26 G venous catheter (BD Neoflon, Heidelberg, Germany) to clear the liver from all intravascular blood, using 10 mL of distilled water (dH_2_O). After removal of blood, livers were resected and the gall bladder removed. A small biopsy was taken for DNA analyses and immune/histological staining (see below). Livers were rinsed and stored in dH_2_O. For physical cell rupture, the livers underwent 2 freeze (−80°C) and thaw (room temperature) cycles. Washing was performed in between and after cycles with dH_2_O for at least 3 × 10 min on an orbital shaker at 200 rpm or until no more cell debris could be removed. Then, another biopsy was taken for DNA analyses and immune/histological staining. Enzymatic proteolysis with a disruption of cell-ECM adhesion was achieved by incubation of the liver in 0.05% Trypsin (Gibco, Paisley, United Kingdom)/0.02% ethylendiamintetracyanat (EDTA; Roth, Karlsruhe, Germany) for 1 h at 37°C (Crapo et al., 2011), which was followed by another washing step and sample biopsy as described above. Next, we applied one of three different chemical decellularisation protocols, using a variety of non-ionic, ionic and acidic solutions at varying concentrations and durations as previously published (De Kock et al., 2011; Kajbafzadeh et al., 2013; Jiang et al., 2014). Some of the livers were incubated in non-ionic detergent 2% (v/v) Triton-X-100 (Sigma Aldrich, St. Louis, Missouri, United States)/0.05% (w/v) EDTA in phosphate buffered saline (PBS, Gibco), whilst others were incubated in ionic detergent 1% (w/v) sodium dodecyl sulphate (SDS; Roth, Karlsruhe, Germany) in PBS or in 0.05% (v/v) ammoniumhydroxide (NH_4_OH; Fluka, Seelze, Germany)/0.5% (v/v) Triton-X-100 in PBS. All incubations were done on a rotator at 20 rpm at 4°C for 36 h and solutions changed at 2, 8, and 20 h. Washing was performed and biopsies were taken at the before mentioned time points of solution changes and after chemical decellularisation. Following macroscopically successful decellularisation (full transparency) or if no further changes of the liver could be detected, livers were removed from the respective solutions. After chemical decellularisation, the cleavage of remaining DNA strands ensued in 1 U/mL RNase-free DNase I (Qiagen, Hilden, Germany) in DNase reaction buffer (Tris-HCl 10 mM, MgCl_2_ 2.5 mM, CaCl_2_ 0.5 mM, pH 7.5) for 2 h at 37°C. Remaining DNase was rinsed by above described washing steps and a biopsy for DNA analyses was taken.

The decellularised liver matrix (DLM) was then sectioned into small cubes of 5 mm × 5 mm × 5 mm using a commercially available egg slicer and then sterilised for 2 h in 0.1% peroxyacetic acid (PAA, Sigma Aldrich) and 4% ethanol in PBS at room temperature, rinsed in three washing steps under sterile conditions with PBS and 2% Penicillin/Streptomycin (Pen/Strep Invitrogen, Waltham, MA, United States), and then stored in sterile PBS and Pen/Strep at 4°C until required.

### 2.2 DNA analyses

Biopsies were taken after liver resection, cryopreservation, enzymatic proteolysis, chemical decellularisation and DNA cleavage, respectively. The samples were dried at 60°C overnight and the dry weight for each sample was documented. Then, each sample was incubated in 5 µL Proteinase K (10 mg/mL, Sigma Aldrich) in 100 µL Sodium-Tris-EDTA (STE) buffer (Tris 1 M, NaCl 5 M, 10% SDS, EDTA 0.5 M, pH 8.0) at 60°C for 2 h. Cell debris was removed by centrifugation at 10,000 rpm for 10 min and DNA precipitated with 100% isopropanol. The DNA was washed with 70% ethanol and dissolved in 5 μL dH_2_O at 60°C. DNA quantification was performed using a Nanodrop ND-1000 (Peqlab, Erlangen, Germany). Measured DNA quantities were related to the respective dry weight to obtain the amount of DNA in nanograms per milligram dry weight.

Gel electrophoresis was used to determine DNA length after the last step of the decellularisation protocol. Gels were prepared with 1.5% agarose in Tris-Borate-EDTA (TBE) buffer and 0.004% Midori Green (Nippon Genetics, Düren, Germany). Samples were supplemented with 6 x DNA loading dye and loaded onto the gel and a 100 bp DNA ladder (GeneRuler, Thermo Fisher, Waltham, MA, United States) was used as marker. Electrophoresis was performed for 40 min at 80 V and the gel images were captured under ultra violet light using a gel documentation system (Intas, Göttingen, Germany).

### 2.3 Cell culture

Four cell lines were used to generate the 3D liver cancer models. HepT1 (Pietsch et al., 1996) and HUH6 (Japanese Collection of Research Bioresources, Osaka, Japan) are HB cell lines, HepG2 (ATCC, Manassas, United States) is a cell line of a HB with hepatocellular carcinoma (HCC)-like features (López-Terrada et al., 2009), and HUH7 is a well differentiated HCC cell line (He et al., 1984; Alexander, 1987). All cell lines were cultured in RPMI 1640 (Life Technologies, Carlsbad, CA, United States), supplemented with 10% (v/v) fetal bovine serum (FBS, Life Technologies) and 1% Pen/Strep in a humidified chamber containing 5% CO_2_ at 37°C.

### 2.4 Generation of 3D tumour models

Sterilised DLMs were incubated overnight in medium at 37°C to prepare the DLMs for seeding on the following day. Cell attachment was ensured either by the drop-on method or the suspension method. In the drop-on technique, 5 µL of medium containing 200,000 cells were added directly onto the DLM, which was then placed in an empty 24-well plate. To allow for cell attachment, the DLMs were kept in a humidified chamber at 37°C for 24 h. For cell seeding using the incubation method, the DLMs were incubated in a suspension of 200,000 cells/mL in low adhesion flasks (Sarstedt, Nürnbrecht, Germany) for 24 h in a humidified chamber at 37°C. The 3D tumour models were then transferred to 24-well plates and to 24-well plates coated with 500 µL of 1.5% (w/v) agarose in dH_2_O, to inhibit cell adherence to the well surface. The models were cultured either attached to the well floor or floating in the well. Attachment was achieved in the agarose-coated 24-well plates by positioning the DLM in the liquid agarose shortly before consolidation. A complete submersion was prevented manually. In the uncoated 24-well plates, the DLMs were positioned on the well floor and media was added after 2 h, when the DLMs had attached. Media changes were performed every 2–3 days. In order to compare the different cell seeding approaches, the percentage of the well surface covered by cells after cell seeding was calculated using brightfield mircroscopy (well coverage in % = area covered by cells in µm^2^/total well surface area in µm^2^ x 100). Then, the repopulation area was measured using DAPI stained cross sections of the hepatoblastoma models (repopulation area in % = area covered with DAPI-positive cells in µm^2^/total DLM area in µm^2^ x 100). For most efficient cell seeding, minimal cell adhesion to the well surface with maximal repopulation of the DLM was aimed at.

### 2.5 Cryopreservation of 3D tumour models

We aimed to find an adequate preservation technique to store ready-to-use tumour models. Firstly, sterilised and dry DLMs without cells were frozen. Secondly, individual tumour models with cells were frozen in 1 mL of freezing medium (10% dimethyl sulfoxide, DMSO (Roth, Karlsruhe, Germany), 40% RPMI 1640, and 50% FBS) in 1.8 ml round bottom cryogenic vials (Nunc, Langenselbold, Germany) on day 1 (d1, directly after cell attachment, before transfer into 24-well plates), day 7 (d7), day 14 (d14) and day 21 (d21) of cultivation. Thirdly, to simulate large-scale applications and reduce transfer steps, multiple models with cells were frozen directly in agarose-coated 24-well plates in freezing medium on d14 and d21 of cultivation (d14 plate and d21 plate). For the cryopreservation of models in their respective 24-well plates, 1 ml of freezing medium was added to each well. Then, the plates were transferred without tilting to a −80°C freezer and subsequently to liquid nitrogen tanks for gradual freezing. All models were kept in liquid nitrogen for a minimum of 3 weeks before revitalisation. All models were thawed and immediately washed in PBS and then in RPMI for 3 × 5 min to remove freezing medium. Tumour models frozen in cryogenic vials were then transferred into a freshly agarose-coated 24-well plate and cultivated until day 21. Models frozen in 24-well plates were thawed and washed in their respective wells. To ensure complete removal of all freezing agents, media changes were performed daily for the first 3 days. Sterilised DLMs without cells were also thawed and then seeded with cells and cultivated for 21 days as described above.

### 2.6 Histology

Liver tissue was fixed in 4% (w/v) paraformaldehyde (PFA, Sigma-Aldrich) in PBS (Gibco) solution for 4 h. DLM samples and 3D tumour models were first embedded in 1.5% (w/v) agarose and then fixed in 4% (w/v) PFA in PBS solution for 4 h. All samples were washed with PBS and dehydrated through a series of ethanol washes (50%, 70%, 90%, and 100%, 2 × 30 min each). This was followed by incubation in ROTI^®^Histol (Roth, Karlsruhe, Germany) for 30 min, in 50% (v/v) ROTI^®^Histol in paraffin (McCormick Scientific, St. Louis, MO, United States) for 2 × 60 min and in pure paraffin overnight. Samples were embedded in paraffin blocks and cut into sections of 5 µm thickness using a Leica SM 2000R microtome (Wetzlar, Germany). Slides were deparaffinised in two changes of ROTI^®^Histol and rehydrated using descending ethanol washes. Hematoxylin and Eosin (H&E) staining on sample sections was performed using Mayers Hematoxylin (Roth) and Eosin (Sigma-Aldrich). Connective tissue was stained using the Elastica van Gieson kit (Morphisto, Offenbach, Germany).

### 2.7 Immunofluorescence

Antigen retrieval was obtained by cooking slides of PFA-fixed and paraffin-embedded samples for 20 min in citrate buffer (citric acid 10 mM, 0.05% Tween, pH 6.0). Slides were incubated for 1 h at room temperature in non-specific blocking buffer (10% goat serum, 0.4% Triton-X-100 in PBS). For fibronectin and collagen IV staining of the murine DLM, rabbit anti-fibronectin polyclonal antibody (dilution 1:400, Invitrogen, MA, United States) and rabbit anti-collagen IV polyclonal antibody (dilution 1:200, Invitrogen) were used as primary antibodies, respectively. Alpha-fetoprotein (AFP) is a specific hepatoblastoma marker and was stained using rabbit anti-AFP polyclonal antibody (dilution 1:500, Acris, Rockville, MD, United States) as a primary antibody. Incubation of the primary antibodies was performed in a humidified chamber at 4°C overnight. Alexa fluor 488-conjugated and Alexa fluor 647 anti-rabbit IgG (Invitrogen) were used as secondary antibodies with 1:1000 dilution. Slides were incubated in a humidified chamber for 1 h and counterstained with 4’, 6-diamidino-2-phenylindole (DAPI, Thermo Fisher).

For DAPI staining of the 3D tumour models *in toto*, media was removed from the well and 200 µl of DAPI/methanol solution (1 μg/ml) were added. After an incubation of 10 min, the 3D tumour models were washed with methanol.

For Live/Death staining, we incubated tumour models simultaneously with 1 µM Calcein-AM (BioLegend, San Diego, CA, United States) as a viability marker, 2 μg/ml Propidium Iodide (PI, Sigma-Aldrich) as a death marker, and 10 μg/ml Hoechst 33342 (Thermo Fisher) as a nuclear counterstain for 20 min at 37°C. Tumour models were washed two times with PBS.

Images of immunofluorescent stainings were captured with the EVOS™ M7000 imaging system (Invitrogen).

### 2.8 MTT cell viability assay

Cell viability was measured using the 3-(4,5-dimethylthiazol-2-yl)-2,5-diphenyltetrazolium bromide (MTT) (Sigma-Aldrich, St. Louis, MO, United States) assay. Tumour models of HepT1, HepG2, HUH6 and HUH7 were generated, cultivated for 14 days, frozen for 1 week in liquid nitrogen, thawed and cultivated for another 7 days. Then, all 3D models were treated with 5 µM cisplatin and 1 µM doxorubicin (both from Selleck Chemicals, Planegg, Germany, and dissolved in DMSO) or the solvent DMSO as a negative control for 72 h. After treatment, 500 µL of MTT1 solution (5 mg/ml MTT in PBS, sterile-filtered) in serum free DMEM (Life Technologies) were added to each well and incubated at 37°C for 4 h. Then, 500 µl of MTT2 solution (10% SDS, 0.37% HCl) were added and the models were incubated overnight at 37°C. After incubation, 150 µL of supernatant were transferred from each well into a 96-well plate and absorbance values were measured using the Sunrise plate reader (Tecan, Männedorf, Switzerland).

### 2.9 Statistical analysis

Statistical analysis was carried out using Microsoft Excel 2019 (Microsoft Corporation, Redmont, United States) and GraphPad Prism 8.2.1.0 software (GraphPad Software, San Diego, CA, United States). Data was non-normally distributed and non-parametric tests were used. Data are presented as violin plots or box plots, indicating the mean ± standard error of the mean. Significance was calculated using the Wilcoxon-rank sum test or the Mann-Whitney *U*-Test. For all tests *p*-values were considered significant at *p* < 0.05 and all statistical tests were two-tailed. Missing data was reported.

## 3 Results

### 3.1 Generation of a decellularised liver matrix (DLM)

**FIGURE 1:**
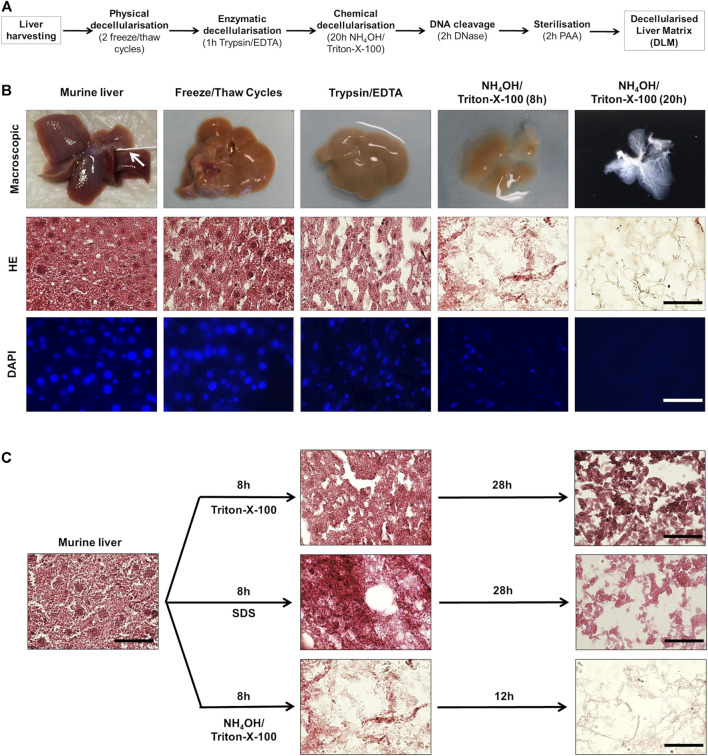
Generation of a decellularised liver matrix (DLM). **(A)** Schematic overview of matrix generation steps. **(B)** Murine liver tissue at different steps of matrix generation. The arrow indicates the venous catheter. The scale bar for H&E and DAPI staining is 50 µm. **(C)** Comparison of different chemical decellularisation techniques. H&E-stained murine liver tissue directly after harvesting and after indicated time (8, 20, and 36 h) of chemical decellularisation in 2% Triton-X-100/0.05% EDTA, 1% SDS, and 0.05% NH_4_OH/0.5% Triton-X-100. The scale bar is 50 µm.

In order to generate a 3D model of hepatoblastoma, we aimed at growing tumour cells on a DLM. Our strategy started with freshly resected murine liver tissue, which was processed through a sequence of physical, enzymatic and chemical decellularisation steps (Figure 1A). Physical decellularisation by freeze and thaw cycles did not show any macroscopic changes to the liver, although murine hepatocytes presented a looser structure and more intercellular gaps in H&E-stained tissue sections (Figure 1B). Incubation in trypsin/EDTA led to a paler appearance of the liver tissue and a loss of cell-cell attachment in the H&E-stain. After testing three different chemical decellularisation protocols, decellularisation by immersion in NH_4_OH in Triton-X-100 solution proved to be most effective regarding decellularisation (Figure 1C). In addition, as decellularisation of the liver *in toto* by immersion was time consuming, the liver was separated into lobes and cut into ready to use 5 mm × 5 mm × 5 mm cubes after cryopreservation using a standard kitchen ware egg slicer.

During decellularisation, the liver tissue showed increasing transparency, as cell debris was rinsed from the connective tissue and a translucent matrix with visible vascular and ECM structures remained (Figure 1B). H&E-staining showed a stepwise decrease of cytoplasmic and nuclear remnants. Correspondingly, immunofluorescent staining of DNA by DAPI showed a decrease of nuclei (Figure 1B).

**FIGURE 2:**
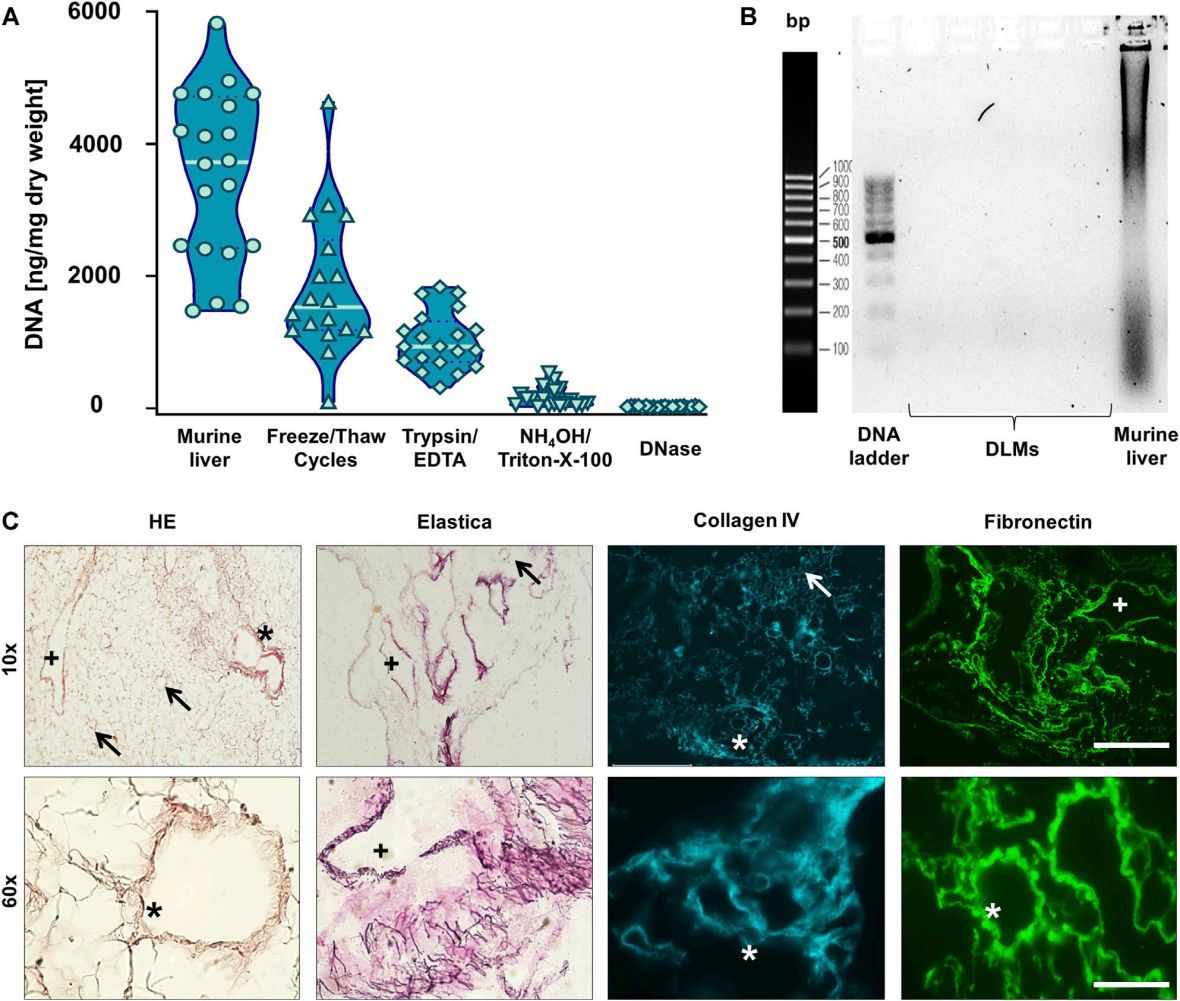
Characterisation of Decellularised Liver Matrix (DLM) **(A)** DNA quantification of liver samples after each decellularisation step. Mean ± standard deviation, *n* = 20. **(B)** Analysis of the length of the remaining DNA in the DLMs as well as murine liver as a positive control after DNase I digestion. **(C)** H&E and Elastica van Gieson stainings as well as collagen IV and fibronectin immunofluorescence of the final DLM. An arrow indicates central veins, a plus indicates larger portal veins, and an asterisk indicates portal triads with portal vein, adjacent biliary duct and hepatic artery. Stainings are depicted in 10- and 60-fold magnification. The scale bars are 500 and 50 μm, respectively.

However, by measuring the DNA content during the decellularisation process, we found that even in livers that appeared completely translucent with no evidence of DAPI-positive nuclei, DNA was still detectable (Figure 2A). Thus, we added an incubation of liver samples with DNase as an additional step into our protocol (Figure 1A). This resulted in a reduction of the DNA amounts from 3,524 ± 1,280 ng/mg dry weight in normal liver tissue to 25 ± 11 ng/mg dry weight in DLM (Figure 2A). Moreover, gel electrophoresis demonstrated that after DNA digestion no DNA longer than 200 bp remained (Figure 2B), which has been defined as a prerequisite in decellularisation protocols (Crapo et al., 2011).

To further characterise the obtained DLM, we first performed H&E–staining, showing an absence of basophilic cell nuclei and a network of eosinophilic staining typical of collagen (Figure 2C). In addition, we used Elastica van Gieson staining which highlighted a web of elastic fibres. Subsequent immunostaining for collagen IV and fibronectin revealed that the basement membrane of the ECM and its structural integrity had been retained by the decellularisation process. In all stainings, the ECM formed a web like structure with retained liver sinusoids draining into central veins and the remnants of portal triads (portal veins, hepatic arteries and biliary ducts), leaving gaps of 15–20 µm diameter were the hepatocytes had resided.

Altogether, our strategy clearly demonstrates that the generation of a cell-free and structurally preserved liver scaffold of murine livers is feasible.

### 3.2 Establishment of a 3D hepatoblastoma model

**FIGURE 3:**
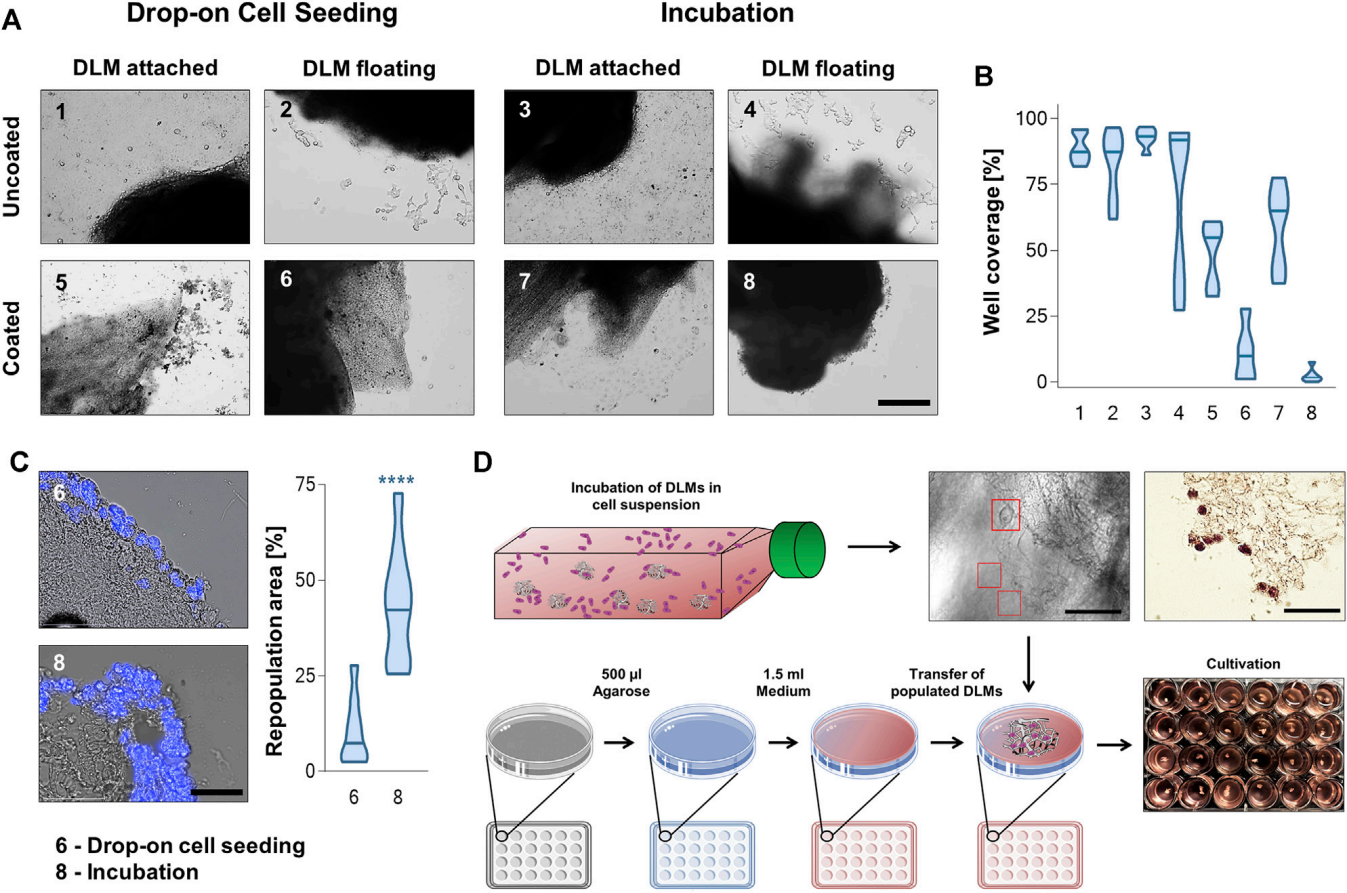
Well preparation, cell seeding and cultivation techniques. **(A)** Brightfield microscopyc pictures of DLMs in uncoated and agarose-coated wells after incubation and drop-on cell seeding protocols. The scale bar is 500 µm. **(B)** Violin plots of the different cell seeding and cultivation approaches in **(A)** depicting the percentage of well surface covered with cells. The plots show the mean and distribution of calculated well coverage. Least well coverage was obtained by cultivating the model floatingly in a coated well (6 and 8) *n* = 6. **(C)** Comparison of DAPI stained cross sections used to calculate the repopulation area after drop-on and incubation cell seeding for set-ups 6 and 8 (both coated wells and floating cultivation). The scale bar is 50 µm. The violin plot shows the percentage of repopulated area of total DLM area. Significance was calculated using the Mann-Whitney *U*-Test ****p < 0.0001. **(D)** Schematic overview of the 3D hepatoblastoma model establishment. DLMs were incubated for 24 h in a low adhesion flask containing 200,000 tumour cells per mL of media. After cell attachment to the matrix (see red boxes in brightfield microscopic image and brown cells in H&E-stain; scale bars are 50 µm), populated DLMs were transferred to wells of a 24-well plate that had been coated with 1.5% agarose to prevent cell adhesion.

**FIGURE 4:**
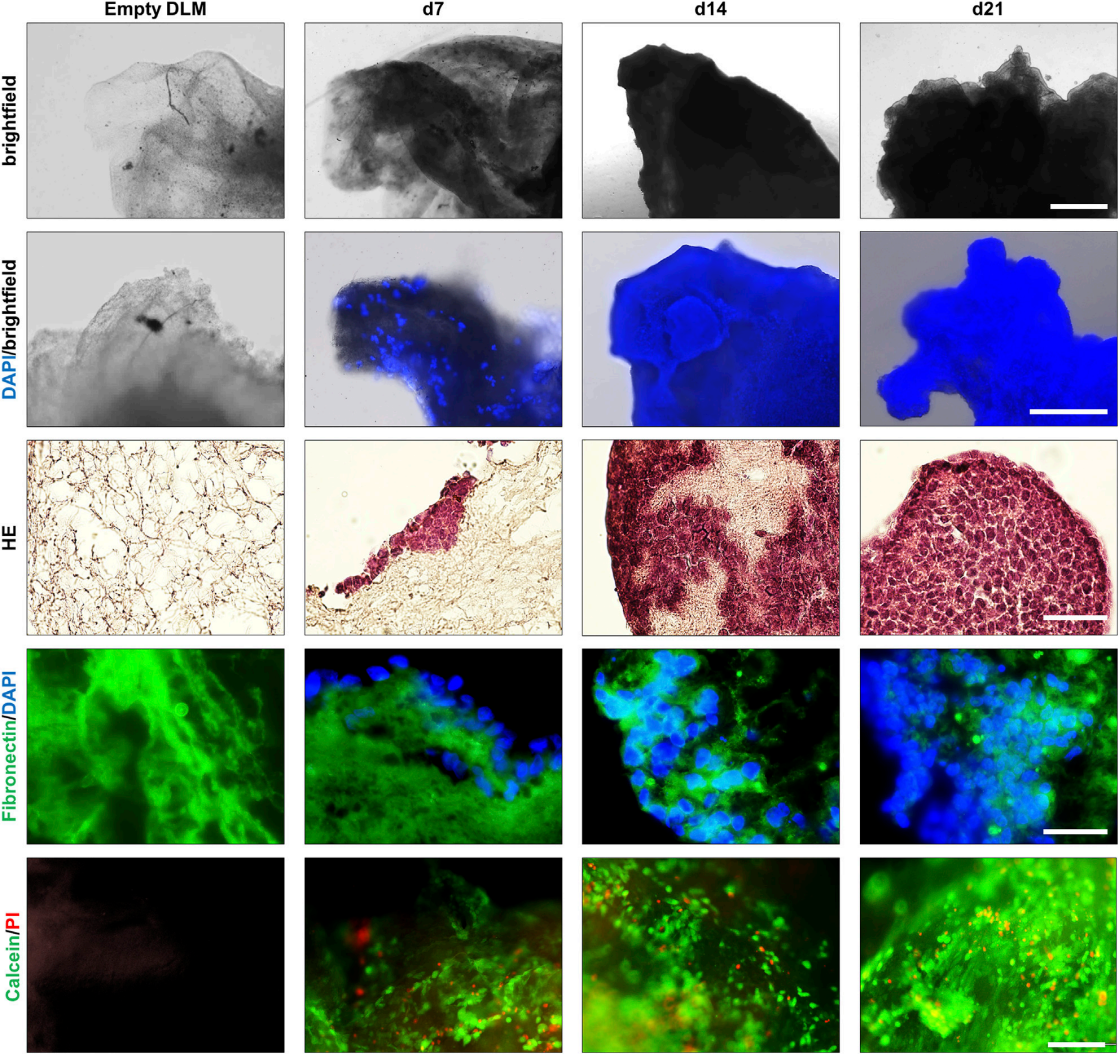
Growth of a 3D hepatoblastoma model over 3 weeks. Images taken of an empty DLM and 7, 14, and 21 days after seeding of HepT1 cells. Population of the DLM and a subsequent cell expansion can be observed between days 7 and 21. Top row: light microscopy images of three dimensional hepatoblastoma models. The scale bar is 1 mm. Second row: Overlay of light microscopy and DAPI staining of three-dimensional hepatoblastoma models. The scale bar is 500 µm. Third row: H&E staining of cross sections of the hepatoblastoma models. The scale bar is 50 µm. Fourth row: Overlay of fibronectin (green) immunofluorescence and DAPI (blue) staining of cross sections of hepatoblastoma models. The scale bar is 50 µm. Bottom row: Live/Death staining of three-dimensional hepatoblastoma models. Overlay of cell viability dye Calcein AM (green) and the death dye propidium iodide (PI, red). The scale bar is 200 µm.

**FIGURE 5:**
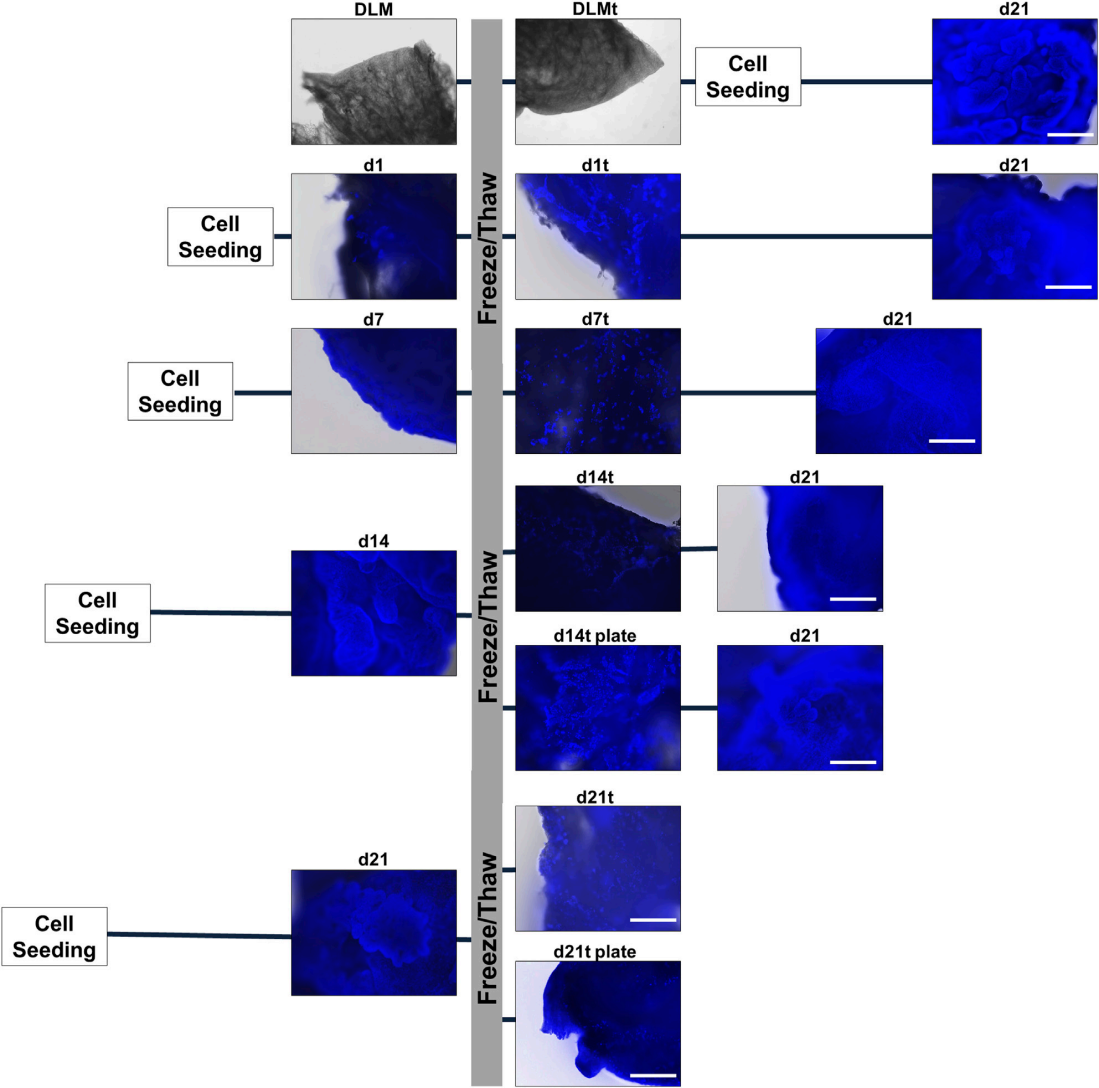
Cryopreservation of 3D hepatoblastoma models. DAPI/methanol staining of hepatoblastoma models directly before freezing (DLM, d1, d7, d14, d21), directly after thawing (DLMt, d1t, d7t, d14t, d14t plate, d21t, d21t plate) and at the end of completed cultivation (d21). The scale bar is 500 µm.

**FIGURE 6:**
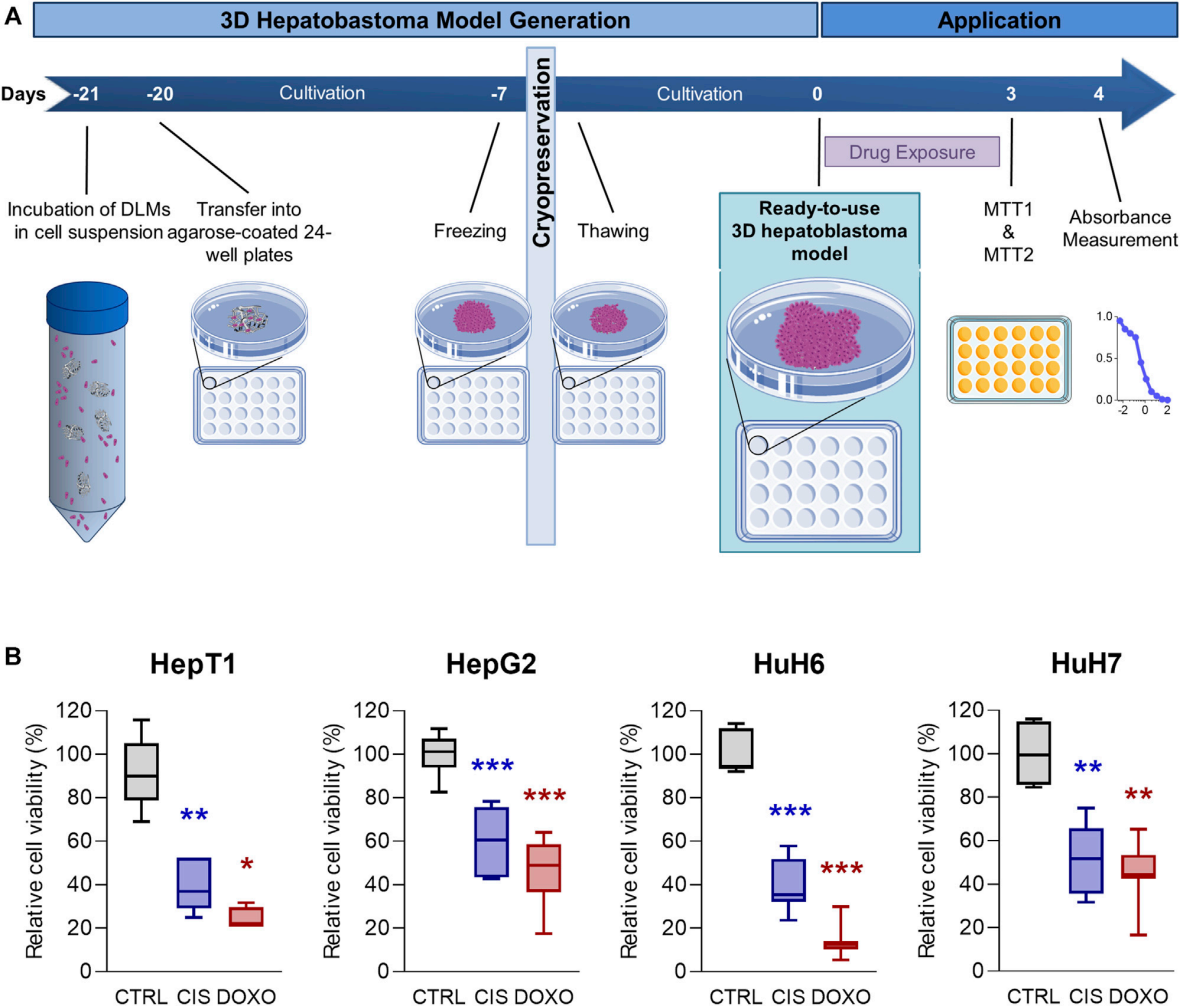
Drug testing on 3D hepatoblastoma models. **(A)** Schematic overview of the 3D hepatoblastoma model generation, preservation and drug testing. 3D tumour models are mass-produced, cultivated for 14 days and cryopreserved in their respective wells, thawed for experimentation, cultivated for another 7 days, and then used for drug testing using MTT assays. **(B)** Cell viability in 3D hepatoblastoma models after chemotherapy treatment. 3D tumour models of HepT1, HepG2, HUH6 and HUH7 (*n* = 4–8) were treated with DMSO (CTRL), 5 µM cisplatin (CIS), and 1 µM doxorubicin (DOXO) for 72 h. Viability was measured using MTT assay. Significance was calculated using the Mann-Whitney *U*-Test (*p < 0.05, **p < 0.01, ***p < 0.001, *n* = 4–8).

In a next step, we used the DLM to establish a 3D hepatoblastoma model. To do so, we tested eight different seeding approaches to populate the DLM with cells; drop-on cell seeding and the incubation of cells together with DLM, both either with the DLM attached to or floating in the well of the cell culture plate and with agarose covered wells or uncoated wells. In the drop-on technique, cells were added directly to the DLM with a pipette, while for the incubation technique, DLMs were incubated in a cell suspension overnight. Cultivation of hepatoblastoma models in uncoated wells showed cell growth mostly on the surface of the well, but not on the DLM, irrespective of which seeding method was used (Figures 3A, 1–4). More than 50%–100% of the well surface was covered with cells for these set-ups (Figure 3B). The same could be seen for the cultivation of hepatoblastoma models that had been attached to the well floor, even if the well had been coated with agarose (Figures 3A, B, 5, 6). To compare the effectivity of the two seeding techniques, DAPI stains of cross sections were evaluated and the repopulated area as percentage of the total DLM area was calculated. Drop-on cell seeding led to a repopulation area of 10.4%, while the incubation technique led to a repopulation area of an average of 43.8%. Therefore, cultivation of the 3D hepatoblastoma models proved more effective when floating in an agarose-coated 24-well plate (Figure 3A), with better cell attachment by incubation than by drop-on cell seeding. Thus, cell seeding by incubation and further cultivation of the models in suspension in agarose-coated wells was chosen as the most efficient procedure (Figure 3B). For large-scale hepatoblastoma model production, the DLMs were incubated in a cell suspension as mentioned before, but in agarose-coated 50 mL falcons instead of low attachment flasks.

Once the cultivation parameters were established, 3D hepatoblastoma models were cultivated over a 3 week period (Figure 4, exemplified for HepT1 cells). Light microscopy showed a loss of transparency of the DLM with continuing tumour cell growth (Figure 4, top row). A DAPI/brightfield overlay as well as subsequent H&E-staining of DLM sections indicated that single tumour cells (DAPI/H&E-positive) started to attach to the surface of the DLM at day 3 (not shown) and that growth of tumour cells into the matrix started at day 7 (Figure 4, 2nd and 3rd row). Cell growth increased gradually from superficial tumour cell nests on the DLM, the population of the entire DLM to the formation of complete 3D structures at day 21 that are vital, as evidenced by Live/Death staining (Figure 4, bottom row). Subsequent immunofluorescent staining clearly demarcated areas of human tumour cells (DAPI-positive) from fibronectin-positive murine DLM (Figure 4). Moreover, staining for the alpha-fetoprotein (AFP), an established marker for liver tumours (Hirohashi et al., 1983), proved the preservation of the disease phenotype of hepatoblastoma (Supplementary Figure S1A).

### 3.3 Cryopreservation of liver tumour models

In order to preserve hepatoblastoma models and stock a large number of ready-to-use models, we tested several cryopreservation procedures. We performed freezing of: i) sterilised DLMs without cells to check for mass production, ii) single established hepatoblastoma models at different developmental stages (d1, d7, d14 and d21) to determine optimal recovery conditions, and iii) ready-to-use d14 and d21 models in agarose-coated 24-well plates to simulate on-demand applications. After 3 weeks in liquid nitrogen, models were thawed and propagated using our established protocol and checked for integrity at different time points.

We observed no difference in the DLM structure before and after freezing, and cell attachment and subsequent cultivation for 21 days proved possible (Figure 5, row 1). Moreover, AFP staining and Live/Death staining proved the manifestation of a vital hepatoblastoma (Supplementary Figure S1). Freezing and thawing of hepatoblastoma models at different time points (d1, d7, d14, d21) and a continuation of cultivation until day 21 was feasible (Figure 5, rows 2–5). However, a considerable loss of nuclei could be seen after freezing and thawing, even in models frozen at the end of cultivation (d21). As expected, the later the models were frozen, the more cells were attached to the DLM before freezing, and the more cells remained attached to the DLM after thawing. However, cells of d1, d7, d14 recovered during the continued cultivation until day 21. Hence, freezing and thawing of the hepatoblastoma models is possible, but involves a temporal throw-back in the cultivation process. Accordingly, DAPI/methanol stains of supernatant freezing-medium and well-plates after the transfer of hepatoblastoma models showed the presence of nuclei and cell debris left in the medium and wells. To minimise cell loss during model transfer and to facilitate large-scale handling, hepatoblastoma models were frozen and thawed in their respective wells in the 24-well plate. This led to more remaining cells after freezing and thawing of the hepatoblastoma models on the DLM and an accelerated regain of 3D structures (d14t plate and d21t plate). Freezing and thawing in the 24-well plates did not however ensure a conservation of 3D structures and a continuation of cultivation remained necessary. Thus, the most time-effective conservation technique is to freeze the hepatoblastoma models at day 14 of cultivation in the 24-well plate and to continue cultivation after thawing until completion of 21 days to allow for regaining characteristic 3D structures.

### 3.4 Testing standard-of-care drugs on the newly established 3D hepatoblastoma models

Having established an optimized protocol (Figure 6A) for the generation of reliable 3D tumour models, we next aimed at using it for preclinical testing of drugs that are currently used as standard-of-care medication in HB patients (Perilongo et al., 2009). After thawing and completing model generation for the cell lines HepT1, HepG2, HUH6 and HUH7, we treated the models with doxorubicin and cisplatin for 72 h and measured cell viability using the MTT assay. We detected a significant loss of cell viability in all 3D tumour models, with higher response rates to doxorubicin than to cisplatin (Figure 6B). Encouragingly, the variance across the experimental replicates of cell viabilities of the individual hepatoblastoma models was low and significance was reached for all cell lines. This demonstrates a high degree of standardisation of our 3D tumour models.

## 4 Discussion

The outcome of high-risk hepatoblastoma (HB) patients still remains poor and novel models reliably replicating features of the disease are urgently needed to investigate new therapy options in an as true to the natural setting as possible (Rikhi et al., 2016). Currently, used cell culture models lack three-dimensional (3D) interactions with extracellular matrix (ECM), while animal models are best for confirmatory end-stage preclinical testing (Huch et al., 2017). The present work describes the generation of new 3D HB models by combining decellularised liver matrix (DLM) and different liver tumour cell lines that can be produced and stored large scale, thereby serving as a reliable preclinical testing platform.

First, we generated a DLM with a preserved structural integrity using an optimised combination of physical cell rupture, enzymatic cell detachment, isotonic stress, DNA cleavage and removal of cell debris with detergents and shear stress (Mazza et al., 2015). The combination of the base NH_4_OH with the non-ionic detergent Triton-X-100 is known to lead to a disruption of nucleic acids, denaturation of proteins and solubilization of cytoplasmic components, while being less damaging to tissue structure than other detergents (Crapo et al., 2011; García-Gareta et al., 2020). This decellularisation procedure proved best to generate DLM from mouse livers with a high cyto-compatibility, which has been defined by: i) no visible nuclei in DAPI or HE stains, ii) <50 ng of DNA per mg of scaffold dry weight, and iii) <200 bp of DNA fragment length (Crapo et al., 2011). While the thorough removal of all cell remnants can occur at the cost of the remaining ECM architecture and biochemical content, our DLM showed a largely conserved 3D structure of the liver ECM proteins collagen IV and fibronectin (Van Der Rest and Garrone, 1991). Previously published data have already shown a preservation of up to 50% of liver specific growth factors in decellularised liver tissue supporting functional hepatocytes, allowing hepatic differentiation, angiogenesis (Soto-Gutierrez et al., 2011), and even proliferation, migration and engraftment of tumour cells into decellularised liver scaffolds (Mazza et al., 2015). As it was shown that liver cells have lesser functional potential if cultivated on collagen gels compared to decellularised liver tissue (Lin et al., 2004), our newly established DLM represent the ideal liver microenvironment to generate HB models allowing complex cell-ECM interactions and the maintenance of cellular functionality (Sellaro et al., 2010; Soto-Gutierrez et al., 2011). This is even superior to the spheroid models that make use of either matrigel or basement membrane extract (Huch et al., 2015; Saltsman et al., 2020), which represented the first step of 3D *in vitro* cultivation of tumour cells with tight cell-cell interactions, but that lack these intricate interactions of tumours with ECM.

Secondly, we established 3D HB models using the four liver tumour cell lines HepT1, HepG2, HUH6 and HUH7 that display different histological and molecular characteristics (Rikhi et al., 2016). The superficial seeding technique by incubation proved best to allow for population of the DLM and formation of 3D structures. All cell lines showed successful adherence to the DLM and a near complete coverage of the surface during the first 2 weeks of cultivation. Longer cultivation times led to the formation of superficial cellular multi-layers and an occupation of the DLM. The standardisation of our protocol for large-scale production established an easy to handle format, while ensuring time- and cost-effectiveness. The standardized size of 5 mm × 5 mm × 5 mm of the DLM, the use of agarose-covered falcons for cell seeding in suspension, and the cryopreservation of HB models in 24-well format allowed for a mass-produced, ready-to-use and on-demand application.

Finally, after successful model generation, mass production and cryopreservation, we aimed to validate our 3D HB model as a drug testing platform. Conducting cell viability assays in the 24-well plate format using the standard-of-care chemotherapies for HB, doxorubicin and cisplatin (Perilongo et al., 2009), showed decreased cell viability in all treated models compared to untreated controls. More importantly, individual models of the same conditions had very small variance in measured data, demonstrating the high degree of standardisation of the models involved. Cell viability was lower in models treated with doxorubicin than in models treated with cisplatin, which is in line with previous data obtained with two-dimensional HB cell culture and spheroids (Eicher et al., 2011). We could replicate these well-known differences in sensitivity using our 3D HB model and are confident that the model will permit future distinction between different drug potencies. Drug testing using our 3D HB models is therefore not only feasible, but also easy to handle, scalable and reliable.

## 5 Conclusion

We established an easy to use, cost- and time-effective three-dimensional hepatoblastoma model based on a specifically generated liver matrix. We could not only mimic characteristic growth of hepatoblastoma cells on and in the matrix, but also the formation of distinctive three-dimensional, vital, AFP-positive tumours. Our models represent the first large-scale three-dimensional testing-platform for childhood liver cancer that can be mass produced and preserved ready for on-demand use, such as drug testing and trial imitation.

## Data Availability

The original contributions presented in the study are included in the article/Supplementary Material, further inquiries can be directed to the corresponding author.

## Supplementary material

SUPPLEMENTARY FIGURE S1 Tumour model functionality and viability shown exemplary for HepG2 hepatoblastoma models. The hepatoblastoma models express the specific hepatoblastoma tumour marker AFP and are viable. **(A)** Immunofluorescence staining of the hepatoblastoma specific tumour marker alpha-fetoprotein (AFP, pink) in cross sections of 3D hepatoblastoma models on day 14 of cultivation and before cryopreservation (top row) and 1 day after thawing (bottom row). Overlay with DAPI staining and DAPI/brightfield imaging. Cryopreservation has no impact on tumour marker expression and viability. The scale bar is 100 µm. **(B)** Live/Death staining for three-dimensional hepatoblastoma models before and after cryopreservation. Overlay of cell viability dye Calcein AM (green) and the death dye propidium iodide (PI, red) as well as Hoechst 33342 (blue) and brightfield. The scale bar is 100 µm.
