# Supplementary figures and images for: Establishing a three-dimensional scaffold model of hepatoblastoma
Source: Front Bioeng Biotechnol. 2023 Nov 23;11:1229490. doi: 10.3389/fbioe.2023.1229490 (PMC10704029; doi:10.3389/fbioe.2023.1229490)

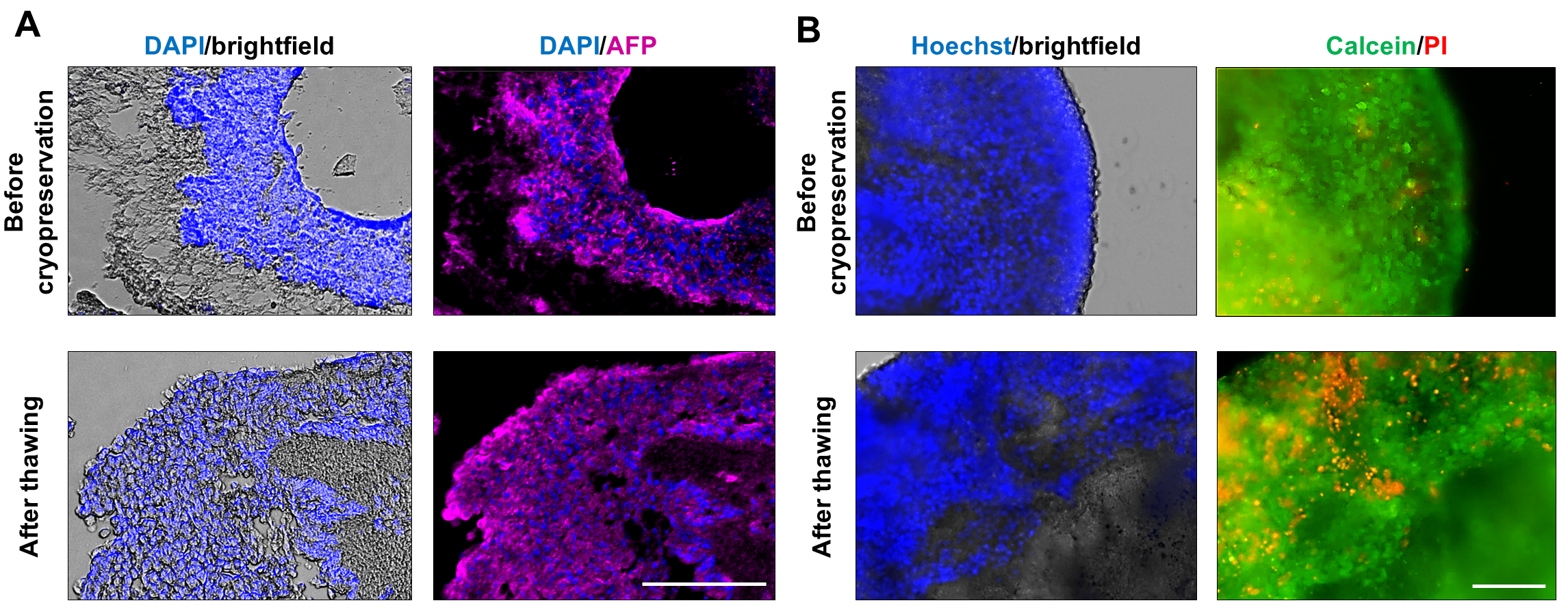

Supplement: Supplementary file 1 [file Image1.TIF]
